# Ergonomic risk and preventive measures of musculoskeletal disorders in the dentistry environment: an umbrella review

**DOI:** 10.7717/peerj.4154

**Published:** 2018-01-15

**Authors:** Simone De Sio, Veronica Traversini, Francesca Rinaldo, Valerio Colasanti, Giuseppe Buomprisco, Roberto Perri, Federica Mormone, Giuseppe La Torre, Fabrizio Guerra

**Affiliations:** 1 U.R. Occupational Medicine, University of Roma “La Sapienza”, Rome, Italy; 2 Specialty School of Occupational Medicine - U.R. Occupational Medicine, University of Roma “La Sapienza”, Rome, Italy; 3 Department of Oral and Maxillo-Facial Surgery, University of Roma “La Sapienza”, Rome, Italy; 4 Department of Public Health and Infectious Diseases, University of Roma “La Sapienza”, Rome, Italy

**Keywords:** Occupational medicine, Preventive measures, Dentistry, Musculoskeletal disorders, Ergonomic risk, Umbrella review

## Abstract

**Introduction:**

Dental practitioners are exposed to different occupational hazards during the course of their professional activity, such as physical, chemical, biological, ergonomic factors. The ergonomic hazards, caused by strained posture and prolonged repetitive movements, can induce musculoskeletal disorders. It occurs in 54–93% of dental professionals and involve the spine, shoulder and hand-wrist tract. Through a systematic review of international literature, we analyzed specific ergonomic risk factors and preventive measures of musculoskeletal disorders in professional dental activity.

**Methods:**

This systematic review is coherent with the PRISMA statement. The scientific research on the major online databases was based on the following keywords: dentist, prevention, ergonomic, dentistry, musculoskeletal, neck pain, posture, ergonomics, work and occupational. The studies included in this review focus on disorders related to ergonomics and on the most effective preventive measures to be adopted. No restrictions were applied for language or publication type. We excluded reports not related to ergonomic prevention in dentistry, reports of minor academic significance, editorial articles, individual contributions, and studies published in scientific conferences.

**Results:**

Online research indicated 4188 references: PubMed (2919), Scopus (1257) e Cochrane Library (12). We excluded 3012 of these, because they were unrelated to ergonomics theme and 187 due to duplication. From the remaining 989 studies, 960 papers did not meet inclusion criteria and they were excluded. Therefore, we analyzed 29 articles, including 16 narrative reviews and 13 original article. The main risk factor for the development of musculoskeletal disorders found in our analysis is static posture adopted during work, highlighted in 87.5% of reviews and 84% of original articles. With regard to preventive measures, 75% of the reviews highlighted the importance of stretching after each working session and at the end of the working day, while 61.5% of the original articles emphasized the use of modern and ergonomic instruments.

**Discussion:**

This review showed that static postures are strongly responsible in the etiology of musculoskeletal disorders. The awkward postures more frequently identified among dental professionals are: extreme forward-head and neck flexion; trunk inclination and rotation towards one side; lifting one or both shoulders; increased curvature of the thoracic vertebral column; incorrect positioning of the lower limbs with thigh-leg angle of less than 90°. It is really important to use of a modern workstation with appropriate ergonomic supports. Among the preventive ergonomic measures, literature has widely recognized the role of physical activity and of a neutral and balanced posture. The present review has some limits: a large part of the selected studies did not have a high methodological quality score and an inadequate statistical analysis.

## Introduction

It is well known that dental practitioners are predisposed to a number of different occupational perils during the course of their professional work (Leggat, Kedjarune & Smith, 2007; Ayatollahi, Ayatollahi & Ardekani, 2012; Gopinadh et al., 2013).

These risks can be classified as: physical, chemical, biological, ergonomic and work-related stress.

Physical threats are due to the use of vibrating tools (Mehta, Gupta & Upadhyaya, 2013), vibration (Rytkönen et al., 2006), non-ionizing (UV) and ionizing (X-rays) radiation.

The chemical risks are the exposure to inorganic agents such as mercury, organic agents such as solvents, resins and anaesthetic gases; caustic agents (formaldehyde and hydrogen peroxide) and allergens (latex) which are also handled (Mehta, Gupta & Upadhyaya, 2013).

The biological hazards may be caused by airborne microorganisms as well as via body-fluid transmission; the most common pathogens are bacteria, viruses (HIV, HBV, HCV) and fungi (Bravo, Correnti & Escalona, 2006; Yonai, 2010).

The work-related stress is due to excessive workloads and can lead to psychological disorders, such as tension, depression, emotional exhaustion and demotivation, all with medico-legal consequences (Ayatollahi, Ayatollahi & Ardekani, 2012).

The ergonomic hazards caused by strained posture and prolonged repetitive movements can induce musculoskeletal disorders (MSDs) (Custodio, Silva & Brandão, 2012; Khalekar et al., 2016).

The most recent study, published in 2005 by Sartorio et al. (2005) reported that these disorders occur in 54–93% of dentists, with a higher incidence in older individuals and women. The areas of the body mainly affected by repetitive strain are the shoulders and wrists, which often display symptoms of carpal tunnel syndrome (Abichandani, Shaikh & Nadiger, 2013; Jodalli et al., 2015).

The most frequently reported disorders among dental practitioners involve the spine, shoulders and hand-wrist tract which can result in lower back pain, neck pain, cervical brachial pain, shoulder tendinitis, De Quervain syndrome, carpal tunnel syndrome and Guyon syndrome (Morse, Bruneau & Dussetschleger, 2010; Biswas et al., 2012; Gupta, Ankola & Hebbal, 2013; Gupta et al., 2014; Tirgar et al., 2015).

Some studies show that MSDs develop over time as a result of incorrect posture at work, and that being aware of these disorders and preventive practices early in professional life can minimize the risk of onset of MSDs. Being vigilant from the early working years can have a lasting impact on the practitioner’s future professional life (Sakzewski & Naser-ud-Din, 2014).

Incorrect posture will lead to MSDs which result in a reduction in productivity and a potential loss of earnings for the dental practice; it is therefore essential that appropriate ergonomic office furniture and equipment should be installed together with appropriate lighting and magnifying instruments that improve visibility (Valachi, 2009; Dable et al., 2014).

The aim of this study was to perform a systematic review of international literature and analysis of specific ergonomic risk factors and preventive measures of MSDs in professional dental activity.

## Materials and Methods

The presentation of this systematic review is in accordance with the PRISMA statement (Moher et al., 2009).

### Literature research

The review included articles published in the last 10 years, from 2006 to 2016 on the major online databases (MEDLINE, SCOPUS, Cochrane Library). The search strategy used a combination of controlled vocabulary and free text terms based on the following keywords: dentist, prevention, ergonomic, dentistry, musculoskeletal, neck pain, posture, ergonomics, work and occupational, used with these search strings: dentist+prevention+ ergonomic, dentistry+musculoskeletal+prevention, dentist+prevention+neck pain, dentistry+posture+ergonomic, ergonomics+dentist, neck pain+dentist, posture+dentist, musculoskeletal+dentist+work, ergonomics+dentistry, work+ergonomincs+dentist, occupational+dentists+ergonomics, musculoskeletal+dentist.

All search fields were considered. Additionally, we practiced a hand search on reference lists of the selected articles and reviews for a wider analysis.

Two independent reviewers (SDS and GLT) performed the search, read the titles and abstracts of the articles identified by the search strategy. Relevant reports were selected according to inclusion and exclusion criteria. Doubts or disagreements were solved through arbitration with a third researcher (FG). Finally, the compatible full texts were independently assessed for definitive eligibility.

### Quality assessment

Three different reviewers assessed the methodological quality of the selected studies with specific rating tools. We used INSA method “International Narrative Systematic Assessment” (La Torre, Backhaus & Mannocci, 2015) to judge the quality of narrative reviews, the Newcastle Ottawa Scale to evaluate cross-sectional and cohort studies (Wells, Shea & O’Connel, 2009); while the JADAD scale was applied for randomized clinical trials (Jadad et al., 1996).

### Eligibility and inclusion criteria

No restrictions were applied for language or publication type. The articles included in this review focus on disorders related to ergonomics and on the most effective preventive measures adopted in the dental profession.

### Exclusion criteria

We excluded articles not concerned with ergonomic prevention or ergonomic risk factors in dentistry, findings of less academic significance, editorial articles, individual contributions (i.e., conferences’ speech), and purely descriptive studies published in scientific conferences without any quantitative and qualitative conclusions.

## Results

Online research indicated 4188 references: Pubmed (2919), Scopus (1257) and Cochrane Library (12). Of these, 3012 were excluded because they were unrelated to dentistry and occupational hazards of dental practitioners. Of the remaining 1176, 187 items were excluded due to duplication.

The full text of 989 studies were assessed, 960 papers that did not meet inclusion criteria were excluded.

**Figure 1:**
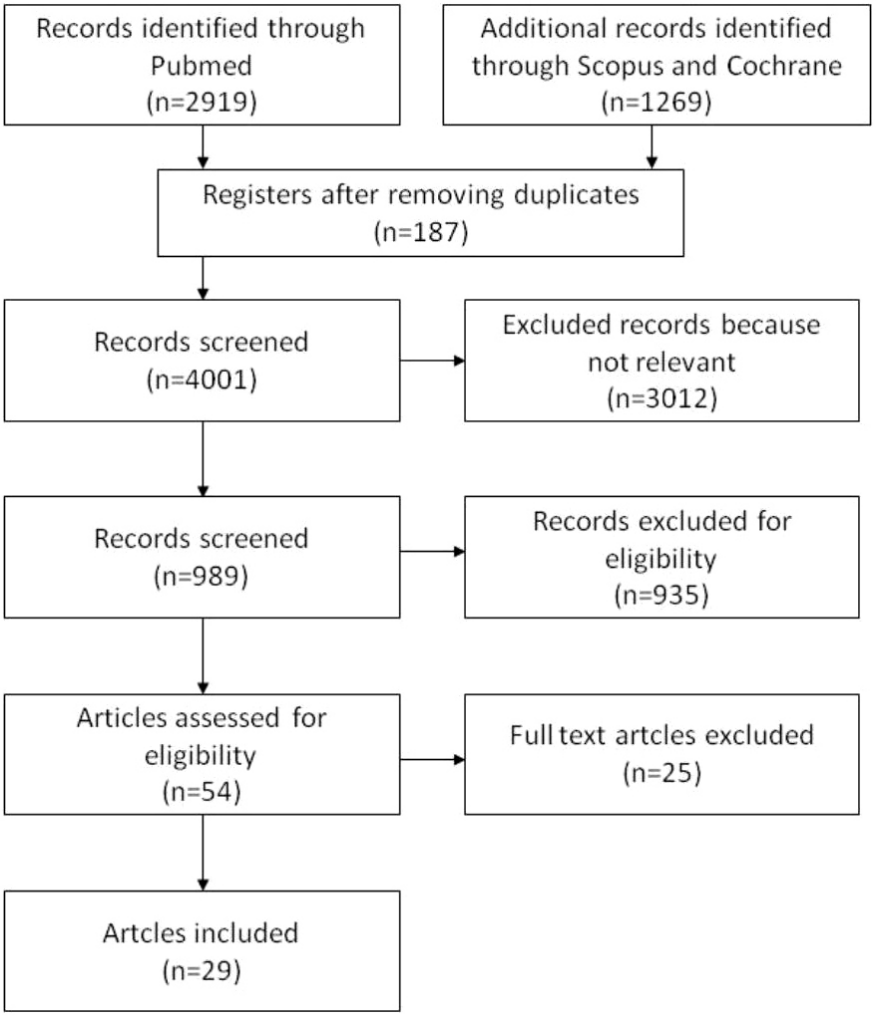
Flow chart for studies selection.

Ultimately, 29 studies were included in this review (Fig. 1). They were 16 narrative reviews and 13 original articles. Among the original articles, 10 were cross-sectional studies, two were clinical trials and one was a case study (Table 1).

**Table 1:** Included studies and methodological quality assessment score.

First author	Study design	Year	Country	Score
AYATOLLAHI J (Ayatollahi, Ayatollahi & Ardekani, 2012)	Narrative review	2012	Iran	3
ABICHANDANI S (Abichandani, Shaikh & Nadiger, 2013)	Narrative review	2013	India	6
ANGHEL M (Anghel et al., 2007)	Cross sectional	2007	Romania	6
CUSTODIO R (Custodio, Silva & Brandão, 2012)	Case study	2012	Brazil	n. a.
DABLE R (Dable et al., 2014)	Clinical trial	2014	India	4
DESAI V (Desai, Pratik & Rajeev, 2012)	Cross sectional	2012	India	8
FINKBEINER B (Finkbeiner & Muscari, 2011)	Narrative review	2011	USA	3
GOPINADH A (Gopinadh et al., 2013)	Cross sectional	2013	India	5
GOSAVI S (Gosavi, Gosavi & Jawade, 2012)	Narrative review	2012	India	3
GUPTA A (Gupta, Ankola & Hebbal, 2013)	Narrative review	2013	India	6
GUPTA A (Gupta et al., 2014)	Narrative review	2014	India	4
GUPTA SHIPRA (Gupta, 2011)	Narrative review	2011	India	3
HADDAD O (Haddad et al., 2012)	Clinical trial	2012	Iran	−1
HARUTUNIAN K (Harutunian et al., 2011)	Cross sectional	2011	Spain	2
HOKWERDA O (Hokwerda, 2008)	Narrative review	2008	Holland	5
JODALLI P (Jodalli et al., 2015)	Narrative review	2015	India	3
KHALEKAR Y (Khalekar et al., 2016)	Narrative review	2016	India	6
KUMAR D (Kumar et al., 2014)	Narrative review	2014	India	5
LEGGAT P (Leggat, Kedjarune & Smith, 2007)	Narrative review	2007	Australia	6
MEHTA A (Mehta, Gupta & Upadhyaya, 2013)	Cross sectional	2013	India	2
MORSE T (Morse, Bruneau & Dussetschleger, 2010)	Cross sectional	2010	USA	7
PEROS K (Peros et al., 2011)	Cross sectional	2011	Croatia	8
PETROMILLI GARCIA P (Garcia, Presoto & Campos, 2013)	Cross sectional	2013	Brazil	3
PIRVU C (Pirvu et al., 2014)	Narrative review	2014	Romania	4
RYTKONEN E (Rytkönen et al., 2006)	Cross sectional	2006	Finland	5
SAKZEWSKI L (Sakzewski & Naser-ud-Din, 2014)	Narrative review	2014	Australia	3
THAKAR S (Thakar et al., 2015)	Cross sectional	2015	India	6
WILLIAMSON R (Williamson, 2015)	Narrative review	2015	USA	1
YAMALIK N (Yamalik, 2007)	Narrative review	2007	Turkey	6

**Notes.**

n.a. not applicable

### Narrative reviews

Regarding the narrative review scores, the INSA score showed an average of 4.18, a median of 4 and a modal value of 3. (Table 1), thus indicating an intermediate quality of the studies. The most appropriate methodological narrative reviews were conducted in Australia, India and Turkey (INSA = 6).

Among risk factors for the development of MSD’s, the most significant is that 87.5% of studies detected static posture during working hours, followed by repetitive movements (68.8% of reviews), muscle imbalances and individual characteristics including a sedentary lifestyle and obesity (43.8%).

Other variables were workplace environment risk factors including: inadequate and non-ergonomic equipment (37.5%), duration and extent of muscle effort (37.5%), vibrating instruments (31.3%) (Table 2).

**Table 2:** Risk factors.

Author	Year	Repetitive movements	Static posture	Muscle imbalances	Workstation^a^	Forceful/ duration	Vibration	Individual Characteristics^b^	Psychosocial factors^c^
**REVIEWS**									
ABICHANDANI S (Abichandani, Shaikh & Nadiger, 2013)	2013	X	X			X	X		
AYATOLLAHI J (Ayatollahi, Ayatollahi & Ardekani, 2012)	2012		X				X	X	
FINKBEINER B (Finkbeiner & Muscari, 2011)	2011				X				
GOSAVI S (Gosavi, Gosavi & Jawade, 2012)	2012		X	X					
GUPTA A (Gupta, Ankola & Hebbal, 2013)	2013	X	X		X	X		X	X
GUPTA A (Gupta et al., 2014)	2014	X	X			X	X		X
GUPTA SHIPRA (Gupta, 2011)	2011	X	X			X		X	
HOKWERDA O (Hokwerda, 2008)	2008								
JODALLI P (Jodalli et al., 2015)	2015	X	X	X	X			X	X
KHALEKAR Y (Khalekar et al., 2016)	2016	X	X	X	X	X			
KUMAR D (Kumar et al., 2014)	2014	X	X	X					
LEGGAT P (Leggat, Kedjarune & Smith, 2007)	2007	X	X					X	X
PIRVU C (Pirvu et al., 2014)	2014		X	X	X				
SAKZEWSKI L (Sakzewski & Naser-ud-Din, 2014)	2014	X	X			X	X	X	X
WILLIAMSON R (Williamson, 2015)	2015	X	X	X					
YAMALIK N (Yamalik, 2007)	2007	X	X	X	X		X	X	X
**ORIGINAL ARTICLES**									
ANGHEL M (Anghel et al., 2007)	2007	X	X	X	X			X	
CUSTODIO R (Custodio, Silva & Brandão, 2012)	2012		X	X					
DABLE R (Dable et al., 2014)	2014		X	X	X				
DESAI V (Desai, Pratik & Rajeev, 2012)	2012	X	X		X			X	X
GOPINADH A (Gopinadh et al., 2013)	2013		X					X	
HADDAD O (Haddad et al., 2012)	2012		X		X				
HARUTUNIAN K (Harutunian et al., 2011)	2011	X	X					X	X
MEHTA A (Mehta, Gupta & Upadhyaya, 2013)	2013	X	X						
MORSE T (Morse, Bruneau & Dussetschleger, 2010)	2010	X	X	X					
PEROS K (Peros et al., 2011)	2011	X	X						X
PETROMILLI GARCIA P (Garcia, Presoto & Campos, 2013)	2013	X	X	X					X
RYTKONEN E (Rytkönen et al., 2006)	2006							X	
THAKAR S (Thakar et al., 2015)	2015							X	

**Notes.**

a tools, temperature, light, magnification, vibration, workload.

b age, sex, BMI, years activity, hobby, genetic, lifestyle.

c stress, organization, demands, control, support, workload, conflict.

Regarding preventive measures, in narrative reviews stretching exercises after each dental examination and at the end of the working day were deemed most useful and effective in preventing muscular disorders (75% of reviews). These were followed by the maintenance of proper, neutral and balanced posture by dental practitioners during examinations (56.3%) and the use of an appropriate workstation in terms of temperature, lighting, and magnifying aids (56.3%).

It should be highlighted that the studies reviewed considered other preventive measures such as the alternation of different postures throughout the working day (31%), the support of a dental assistant with the use of ergonomic instruments (18%), and alternative relaxing techniques as less significant (12%) (Table 3).

**Table 3:** Preventive measures.

Author	Year	Stretching exercises	Short breaks	Ergonomic design^a^	Balanced posture	Proper wokstation^b^	Alternate postures	Instruments^c^	Assistant	Physical activity/ health behaviour^d^
**REVIEWS**										
ABICHANDANI S (Abichandani, Shaikh & Nadiger, 2013)	2013	X		X	X					
AYATOLLAHI J (Ayatollahi, Ayatollahi & Ardekani, 2012)	2012									
FINKBEINER B (Finkbeiner & Muscari, 2011)	2011			X					X	
GOSAVI S (Gosavi, Gosavi & Jawade, 2012)	2012	X	X	X	X		X			
GUPTA A (Gupta, Ankola & Hebbal, 2013)	2013	X		X		X				X
GUPTA A (Gupta et al., 2014)	2014	X		X	X	X		X	X	
GUPTA SHIPRA (Gupta, 2011)	2011	X	X	X	X	X		X		
HOKWERDA O (Hokwerda, 2008)	2008			X	X	X				
JODALLI P (Jodalli et al., 2015)	2015	X		X		X		X	X	
KHALEKAR Y (Khalekar et al., 2016)	2016	X	X	X	X	X	X			
KUMAR D (Kumar et al., 2014)	2014	X								
LEGGAT P (Leggat, Kedjarune & Smith, 2007)	2007	X								
PIRVU C (Pirvu et al., 2014)	2014	X	X	X	X	X	X			
SAKZEWSKI L (Sakzewski & Naser-ud-Din, 2014)	2014	X	X	X	X	X	X			X
WILLIAMSON R (Williamson, 2015)	2015	X		X			X			
YAMALIK N (Yamalik, 2007)	2007			X	X	X				
**ORIGINAL ARTICLES**										
ANGHEL M (Anghel et al., 2007)	2007	X	X	X	X	X	X			
CUSTODIO R (Custodio, Silva & Brandão, 2012)	2012			X	X	X			X	
DABLE R (Dable et al., 2014)	2014			X	X					X
DESAI V (Desai, Pratik & Rajeev, 2012)	2012				X					X
GOPINADH A (Gopinadh et al., 2013)	2013	X		X						X
HADDAD O (Haddad et al., 2012)	2012			X	X	X				
HARUTUNIAN K (Harutunian et al., 2011)	2011	X	X	X		X				X
MEHTA A (Mehta, Gupta & Upadhyaya, 2013)	2013		X							
MORSE T (Morse, Bruneau & Dussetschleger, 2010)	2010	X		X	X					
PEROS K (Peros et al., 2011)	2011	X								X
PETROMILLI GARCIA P (Garcia, Presoto & Campos, 2013)	2013									
RYTKONEN E (Rytkönen et al., 2006)	2006	X	X							X
THAKAR S (Thakar et al., 2015)	2015	X		X						

**Notes.**

a stool, patient’s chair, arm/back supports.

b Light, magnification, temperature, gloves.

c Hand, Authomatic.

d yoga, meditation, sports, relaxation.

### Original articles

The scores assigned to the original articles have an average value of 4.5 and a median value of 5 (Table 1). This demonstrates an intermediate quality of the studies; the studies conducted in Croatia, India and the USA obtained the highest values.

In these articles, static posture appeared to be considered the main cause of MSDs described in over 84% of the studies, followed by repetitive movements (53.8%), individual characteristics (such as sex, age, BMI, lifestyle) (46.2%), muscular imbalances (38.5%), psychosocial factors such as stress, conflict with colleagues, workload and workstation characteristics (30.8%) (Table 2).

In 61.5% of the original articles, the use of modern and ergonomic instrumentation played a key role in preventing MSDs, followed by stretching exercises (53.8%), maintenance of correct posture and healthy lifestyles (46%), taking short breaks after each dental examination and the use of appropriate workstations equipped with adequate magnification and lighting appliances (30.8%); less attention is paid to the alternation, of standing/sitting positions throughout the working day and to the support of an assistant (7.7%) (Table 3). At support of these preventing measures, Peros et al. (2011) revealed that students of Dental course degree who attended the physical fitness course had significantly better physical fitness (*p* = 0.008) than those who did not. Students who exercised more regularly had significantly less low back pain (*r* =  − 0.19, *χ*2 = 11.89, *p* < 0.01) than those who did not.; Also, Gopinadh et al. (2013) denmostrated that students in dentistry that had chosen physiotherapy (39.1%) and yoga (28.6%) as the most important factors to relieve their pai. About the lighthing appliances it should be noted that no experimental studies or cross-sectional ones have been found on this subject. However, literature widely discussed about the essential role of a proper and suitable illumination in order to improve the operator’s field of vision and to ensure that he does not assume incongruous postures during the interventions. According to Valachi (2009), in fact, the operating light should be positioned parallel to the operator’s visibility line, or at most within 15 degrees of inclination; This requires that the light is positioned behind dentist’s head, but that’s often difficult because of the fixed workstations. The use of light-emitting microscopes helps health workers, because aligning the direction of the lamps with the visual field, they prevents the “shadow effect”. Gupta, Ankola & Hebbal (2013) also reported that the ratio of intensity between the light of the operative field and the surrounding environment, should be within the range of 3 to 1.6.

The methodological quality was acceptable in only one of the two trials examined. In this trial (Dable et al., 2014), the authors compared three different groups of thirty dental professionals. Each group was assigned a different type of seating (‘Salli Saddle Chair’, a conventional chair with back rest, and conventional chair without back rest) with and without theuse of magnifying instruments. The body postures were analyzed with RULA “Rapid Upper Limb Assessment” system. The results showed a slight risk of developing MSDs using the “Saddle Chair” with adequate magnification instruments. No differences were found between the two conventional chairs with or without backrest.

In a study by Haddad et al. (2012), 12 volunteers tested a recently designed ergonomic dental chair (EDC) with chest and arm support, during which was recordered an Electromyography (EMG) from the trapezius muscle compared the EDC with an ordinary dentist’s seat. The results show that EDC had a significant (*p* < 0.001) favorable effect on EMG activities of the trapezius.

## Discussion

The rationale for conducting this review was twofold: firstly, the most recent study regarding this matter was published in 2005; secondly, the aim was to conduct a more comprehensive review utilizing innovative methodological tools developed since the last review.

Our review showed that certain risk factors present in the dentistry field deserve proper consideration, particularly for the prevention of MSDs.

Prolonged static posture is considered one of the main causes of MSDs and should be the focus of risk assessment by occupational physicians to facilitate the development of effective preventive strategies (Peros et al., 2011; Roberts, Gallardo & Brown, 2014).

The most frequently observed incorrect postures (Kanteswari et al., 2011) in dental practitioners are:

• Extreme forward head tilt and overstretched neck;
• trunk inclination and rotation towards one side;
• the raising of one or both shoulders;
• increased curvature of the thoracic vertebral column;
• incorrect positioning of the lower limbs with thigh-leg angle of less than 90°.

The forward-head tilt and rounded-shoulder postures increase loads on the upper neck muscles (upper trapezius and levator scapulae) and spinal vertebral discs (Haddad et al., 2012).

Frequent trunk inclining and twisting to one side, is very often caused by incorrect positioning of the workstation, tools and materials. When dental equipment is not at an appropriate working height, distance and position, the dental professional is forced to sustain an unbalanced position (Gupta, 2011). By receiving/handing tools to the dentist, dental assistants can play an important role in reducing repetitive movements and poor posture that can cause MSDs (Finkbeiner & Muscari, 2011; Gosavi, Gosavi & Jawade, 2012).

According to Custodio, due to limited workspaces, the positions recommended by ISO and FDI for both the patient and the professional (respectively supine and at 9 o’clock) are rarely respected (Custodio, Silva & Brandão, 2012).

Furthermore, some authors indicate the importance of both the position of the patient’s chair which should be a elevated in accordance to the dental worker’s height, and the use of a suitable lighting source (Gosavi, Gosavi & Jawade, 2012).

If the dental professional’s seat does not permit a 90°  angle with the knees and does not provide proper lumbar support, the spinal curvature is reduced leading to posterior rotation of the lower limbs. For this reason a workstation with appropriate ergonomic supports is fundamental (Pirvu et al., 2014; Porter, 0000).

Recently, some studies have highlighted risk factors such as obesity and physical inactivity in the development of chronic MSDs in medical practitioners; often due to exhaustion and fatigue caused by a heavy workload and long working hours (Harutunian et al., 2011; Thakar et al., 2015).

The published literature documents the important role played by physical activity, aerobics and stretching as preventive ergonomic measures. Aerobic exercises, as the name suggests, improves the oxygen flow to the tissues, thus increasing efficiency. Stretching exercises are effective in relaxing and reducing muscle tension caused by incorrect posture (Kumar et al., 2014). It is well known that a prolonged static posture needs contraction of 50% of the total body muscles, and this requires stretching the tensed muscles. In order to reduce the tension in the muscles, it is recommended, as suggested some authors (Kendall, McCreary & Provance, 1993; Milerad et al., 1991) a slow, gentle and pain free stretch maintained for 15–30 s, performed 2–3 times a day.

Maintaining a neutral, balanced posture or alternating between different positions during examinations was a major factor in preventing MSDs in dentists which emerged during this literary review (Todd, Bennett & Christie, 2007; Anghel et al., 2007; Gosavi, Gosavi & Jawade, 2012). These results are consistent with a study that shows how dentists working exclusively in a sitting position are more likely to be affected by lumbar pain than those who alternate between sitting and standing (Pirvu et al., 2014).

The correct working posture in dental practice is neutral and symmetrical; the clinician is seated with the pelvis and shoulders parallel, the legs are slightly apart, the trunk perpendicular to the floor, the arms are close to the body, the forearms are horizontal and the head is flexed by 20–25°  (Anghel et al., 2007).

All work related risks and their respective preventive strategies should be explained at the undergraduate training stages, so that students can prevent the onset of MSDs by adopting an ergonomic approach in their dental practice (Garcia, Presoto & Campos, 2013).

The limits of this review are linked to the mediocre methodological quality score and an inadequate statistical analysis of included studies, which were mainly conducted in Asian countries (55%). In addition, the limited number of clinical trials published greatly reduced the evidence base of the results. Therefore, to be able to fully assess risks and preventive factors associated with the MSDs in dentists, further randomized clinical trials are imperative.

In conclusion, to prevent MSDs a targeted healthcare protocol is required following the advice of physiatrists so that the occupational physician, including professional figures such as dentists could benefit. The goal of the protocol should be to prevent the incidence of posture related disorders by indicating clinical and instrumental tests aimed at establishing if workers are exposed to specific risk factors. Furthermore, the protocol should indicate preventive strategies such as the correct posture for dental practitioners and exercises that improve aerobic and physical activity.

##  Supplemental Information

10.7717/peerj.4154/supp-1 Data S1 Raw data

10.7717/peerj.4154/supp-2 Supplemental Information 1 PRISMA checklist

10.7717/peerj.4154/supp-3 Supplemental Information 2 PRISMA flow diagram

## Additional Information and Declarations

### Data Availability

The following information was supplied regarding data availability: The raw data has been provided as a Supplemental File.
