# Supplementary figures and images for: Ergonomic risk and preventive measures of musculoskeletal disorders in the dentistry environment: an umbrella review
Source: PeerJ. 2018 Jan 15;6:e4154. doi: 10.7717/peerj.4154 (PMC5772380; doi:10.7717/peerj.4154)

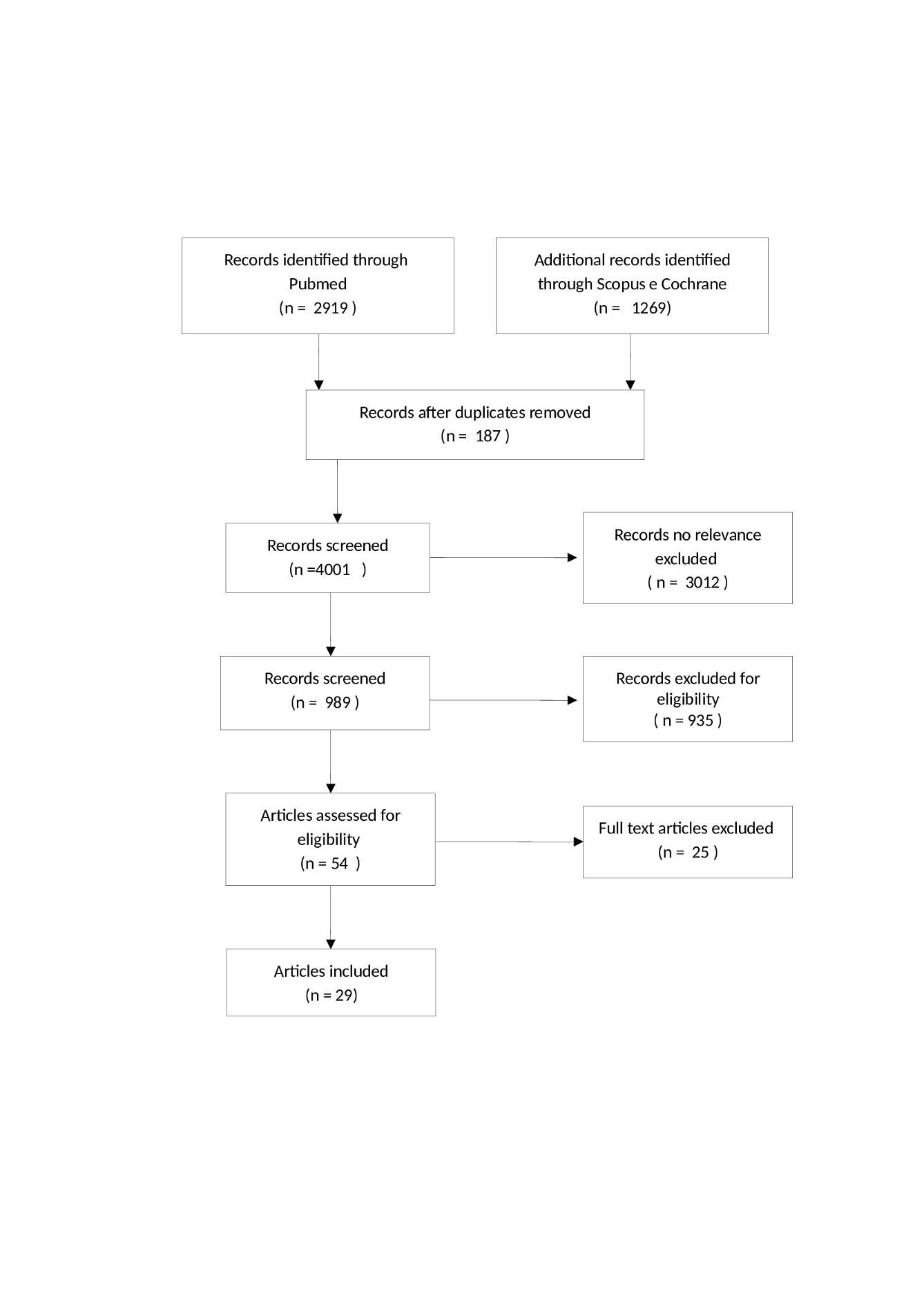

Supplement: Supplemental Information 2 [file peerj-06-4154-s003.doc]
